# *Ureaplasma* antigenic variation beyond MBA phase variation: DNA inversions generating chimeric structures and switching in expression of the MBA N-terminal paralogue UU172

**DOI:** 10.1111/j.1365-2958.2010.07474.x

**Published:** 2011-02

**Authors:** Carl-Ulrich R Zimmerman, Renate Rosengarten, Joachim Spergser

**Affiliations:** Institute of Bacteriology, Mycology and Hygiene, University of Veterinary Medicine ViennaVeterinaerplatz 1, A-1210 Vienna, Austria

## Abstract

Phase variation of the major ureaplasma surface membrane protein, the multiple-banded antigen (MBA), with its counterpart, the UU376 protein, was recently discussed as a result of DNA inversion occurring at specific inverted repeats. Two similar inverted repeats to the ones within the *mba* locus were found in the genome of *Ureaplasma parvum* serovar 3; one within the MBA N-terminal paralogue UU172 and another in the adjacent intergenic spacer region. In this report, we demonstrate on both genomic and protein level that DNA inversion at these inverted repeats leads to alternating expression between UU172 and the neighbouring conserved hypothetical ORF UU171. Sequence analysis of this phase-variable ‘UU172 element’ from both *U. parvum* and *U. urealyticum* strains revealed that it is highly conserved among both species and that it also includes the orthologue of UU144. A third inverted repeat region in UU144 is proposed to serve as an additional potential inversion site from which chimeric genes can evolve. Our results indicate that site-specific recombination events in the genome of *U. parvum* serovar 3 are dynamic and frequent, leading to a broad spectrum of antigenic variation by which the organism may evade host immune responses.

## Introduction

*Ureaplasma* (*U.*) *urealyticum* and *U. parvum* are members of the class *Mollicutes*, commonly referred to as mycoplasmas. Both species are commensals of the human urogenital tract and of newborn infants and are gaining recognition as important opportunistic pathogens during pregnancy (Naessens *et al*., 1987; Gray *et al*., 1992; Cassell *et al*., 1993; Abele-Horn *et al*., 1997; Yoon *et al*., 1998; Vogel *et al*., 2006). There are four distinct *U. parvum* serovars (1, 3, 6 and 14) and 10 *U. urealyticum* serovars (2, 4, 5, 7–13). Genetic analysis has led to the division of human ureaplasmas into these two species on the basis of differences in genome size (Robertson *et al*., 1990), DNA/DNA hybridization (Christiansen *et al*., 1981) and restriction fragment length polymorphisms (Harasawa *et al*., 1991). In addition, the 16S rRNA gene, the 16S–23S rRNA intergenic regions, genes for subunits of the urease gene and the multiple-banded antigen (MBA) gene are used to define species and subtypes (Robertson *et al*., 1993; Knox *et al*., 1998; Harasawa and Kanamoto, 1999; Kong *et al*., 1999; 2000a,b;).

The MBA is a distinct size-variable surface protein expressed by both *Ureaplasma* species and was therefore proposed to play a major role in antigenic variation and virulence (Zheng *et al*., 1995). The *mba* gene consists of a conserved part encoding both a signal peptide and a membrane anchor, and a variable part, encoding a number of uniform repeating units. In *U. parvum* serovar 3, each unit comprises the six amino acids QPAGKE. Size variation of the MBA protein, which occurs both *in vivo* and *in vitro*, is caused by alterations in the number of repeat units (Teng *et al*., 1994; Zheng *et al*., 1994; 1995;), an event that is attributed to slipped-strand mispairing (illegitimate recombination).

A recent report demonstrated how MBA-negative mutants were obtained from *U. parvum* serovar 3 and *U. urealyticum* serovar 5 through selection by applying pressure with MBA-specific antibodies (Monecke *et al*., 2003). Furthermore, it was shown that the *mba* gene is alternating in expression with its adjacent ORF UU376 and that the 5′ region of the *mba* gene, which encodes the potential membrane anchor, can be fused to either ORF via a DNA inversion event presumably occurring at short inversion sequences (Zimmerman *et al*., 2009). We therefore postulated that the two ORFs UU375 and UU376 belong to a gene family composing the *mba* locus.

Aside from the *mba* gene, five additional MBA N-terminal paralogue ORFs (UU172, UU189, UU483, UU487 and UU526) have been annotated in *U. parvum* serovar 3 (strain ATCC 700970) (Glass *et al*., 2000), all of which show high sequence similarity in the 5′ region, which encodes both signal peptide and the potential membrane anchor. Among these five MBA N-terminal paralogues, UU172 and its adjacent ORF UU171 both show striking similarity to the *mba* locus. Both ORFs are oriented in opposite direction and UU171, which was originally annotated as ‘conserved hypothetical’, shows high sequence similarity to UU376 (residues 1–187 are 60% similar to residues 45–224 of UU376). Moreover, two inverted repeats can be located in this region; one in the 5′ sequence of UU172 and another in the intergenic spacer region between UU172 and UU171. The resemblance of this UU172 element to the *mba* locus indicated that it too might undergo alternating expression.

To date, the genomes of the two *U. parvum* serovar 3 strains ATCC 700970 (Accession No. AF222894) (Glass *et al*., 2000) and type strain ATCC 27815^T^ (Accession No. CP000942), and the genome of *U. urealyticum* serovar 10 strain ATCC 33699 (Accession No. CP001184) have been completely sequenced. Moreover, draft assemblies are available for the remaining *U. parvum* and *U. urealyticum* serovars (J. Craig Venter Institute). In all 14 serovars, orthologues of the ORFs UU171 and UU172 exist, and the sequences are highly conserved among them. When aligning these particular genomic regions of all 14 serovars, two major observations can be made: first, an inversion quite similar to the one described for the *mba* locus of serovar 3 can be seen among the mentioned ORF clusters of the serovars and second, a major part of the sequence of the ORF annotated as UU144 in serovar 3 is fused to the equivalent of UU171 in all other serovars. Only in the two sequenced serovar 3 strains are the two ORFs UU171 and UU144 separated/fractionated by a large genomic region of over 20 kb.

Since to date only one sequence is publicly available for each serovar, it was unclear whether the observed inversions of the UU172 element among the serovars had occurred in a common ancestor, or whether it is a frequently ongoing process occurring within proliferating populations. The objective of this study was therefore to provide evidence on both the genetic and protein level, that the proposed phase switching between UU172 and UU171 is an event occurring within defined clonal populations and that the phase switching equally occurs in both *Ureaplasma* species. In addition, our intention was to detect protein expression of the hypothetical ORFs UU171 and UU144.

To avoid confusion and to facilitate reading, the ORFs described in this report were named according to the locus tags defined for *U. parvum* serovar 3 strain ATCC 700970 (Glass *et al*., 2000). As a result of our findings about what we describe as the UU172–UU171–UU144 phase-variable element, these genes have been re-annotated in the ureaplasma genome sequences now in public databases.

## Results

### Organization of the UU172 element in *Ureaplasma* serovars and clonal variants of *U. parvum* serovar 3 isolates

**Fig. 1:**
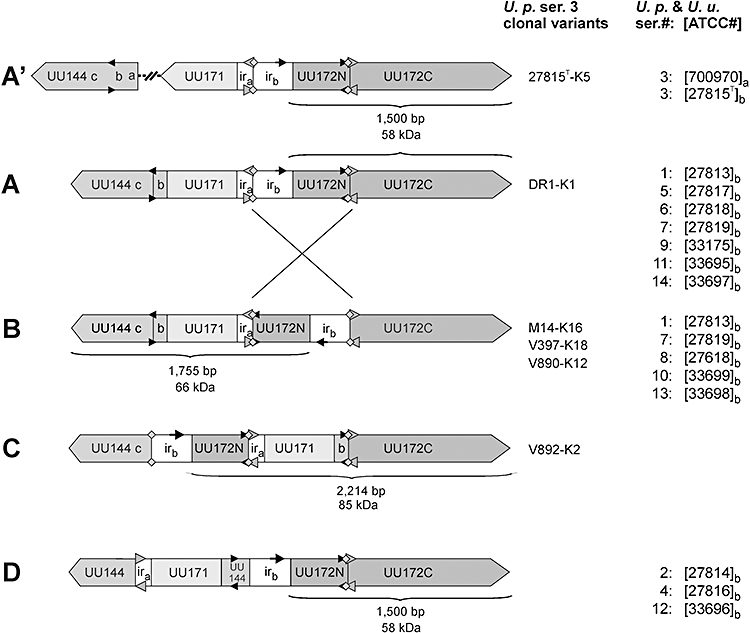
Arrangements of the UU172 element in *U. parvum* and *U. urealyticum*. Schematic illustration of the UU172 element in (A′) *U. parvum* serovar 3 strain ATCC 700970 and strain ATCC 27815^T^ and the corresponding locus in (A–D) clonal variants of clinical isolates of *U. parvum* serovar 3 and other *U. parvum* (*U. p.*) and *U. urealyticum* (*U. u.*) strains [_a_: reference (Glass *et al*., 2000); _b_: sequence information of the J. Craig Venter Institute]. ser.: *Ureaplasma* serovars. Black arrow: potential promoter of the UU172 gene locus. ir_a_ and ir_b_: intergenic spacer regions. Crossing between A and B: suggested DNA inversion. Molecular weights were calculated on the basis of amino acid sequences.

To determine the genomic organization of the UU172 element in *U. parvum* serovar 3 clonal isolates, genomic DNA was extracted from clonal variants of five strains (M14, V890, V397, DR1 and V892), digested with BglII, and their UU172 loci were cloned in *Escherichia coli* and sequenced. In strain ATCC 700970, BglII restriction sites are located in the surrounding hypothetical ORFs UU168 and UU173 and they generate a 6731 bp fragment. None of the investigated strains showed a similar gene arrangement to that of strain ATCC 700970 or strain ATCC 27815^T^. In the five investigated strains, the sequence of UU144 was located adjacent to the sequence of UU171 (Accession No. FN601249, FN601251, FN601252, FN601253, FN601255).

blast analyses revealed that UU144, UU171 and UU172 exist as orthologues in all 14 described *Ureaplasma* serovars (Tables S1 and S2); however, a UU171/UU144 fusion disrupted by a 20 kb region is only present in the two sequenced serovars 3 strains, but in none of the other *U. parvum* or *U. urealyticum* serovars. The five configurations of the UU172 element observed in clinical isolates and in the 14 described serovars are illustrated in Fig. 1.

### Expression of potential membrane proteins encoded in the UU172 element

**Fig. 2:**
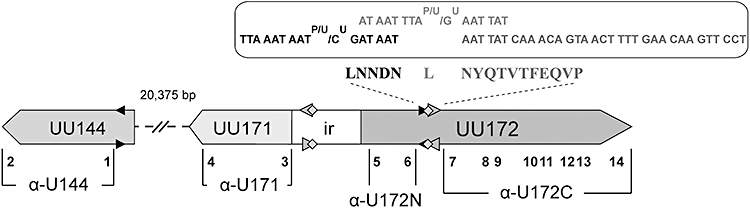
Schematic illustration of the disrupted UU172 element of *U. parvum* serovar 3 strain ATCC 700970 showing the ORFs UU172, UU171 and UU144 and the intergenic region (ir) between UU172 and UU171. Triangles and diamonds represent inverted repeat regions, where the diamond indicates a palindromic sequence; corresponding DNA and encoding amino acid sequences, as they appear in *U. parvum* and *U. urealyticum*, are shown above. ^P/U^: nucleotides found in *U. parvum* and *U. urealyticum* serovars 2, 4 and 12. ^U^: nucleotides in *U. urealyticum* strains except serovars 2, 4 and 12. Primers (designated as numbers) used for generating recombinant proteins and monospecific Pabs used for their detection are indicated (see Table S3).

The findings of the blast analyses and the sequence data indicate that the two ORFs UU144 and UU171 compose one potential reading frame (UU171/144) in the investigated isolates – with the exception of V892-K2 – and together with UU172 comprise one phase-variable locus, where the N-terminal part of UU172 (UU172N) undergoes inversion. To prove this, four recombinant fusion proteins were synthesized (Table S3, Fig. 2) and used to generate monospecific hyperimmune antisera (Pabs) in rabbits for Western blot analyses. Pab α-U172N was generated to detect all possible protein variations before and after the suggested DNA inversion. The recombinant protein U172N comprised 102 amino acids of the N-terminal region of UU172. The first 30 amino acids were omitted, as they encode a potential signal sequence, which is conserved among the five MBA N-terminal paralogues in *U. parvum* serovar 3. Sequences that contained potential DNA inversion sequences within UU172 and UU144 were also omitted to avoid possible cross-reactivity of Pabs in immunoblots.

**Fig. 3:**
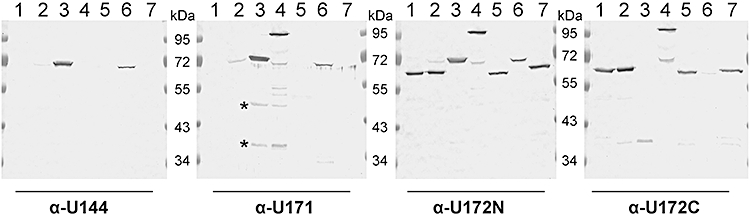
Western blot analyses of total *Ureaplasma* cell protein. *U. parvum* serovar 3 clonal variants: lane 1, 27815^T^-K5; lane 2, DR1-K1; lane 3, M14-K16; lane 4, V892-K2; *U. urealyticum*: lanes 5–7, serovars 9, 10 and 12 respectively. Proteins were detected with monospecific Pabs (see Table S3) indicated below the blots. Asterisks: cross-reactivity with UU376 protein.

The purified recombinant fusion proteins were cleaved from MalE with Factor Xa for use in Western blots to test the reactivity of the Pabs. All proteins were detected by their ‘corresponding’ Pabs which did not cross-react (Fig. S1). Pab α-U171, however, cross-reacted with the protein encoded by UU376 (Fig. S2), due to high sequence similarity. This cross-reactivity was particularly observed in Western blots of total protein of strains M14 and V892 (Fig. 3), which showed a higher expression rate of UU376 than the other strains (Zimmerman *et al*., 2009).

To determine protein expression of the UU172 loci in the investigated strains, all four monospecific Pabs were used for immunostaining of total protein from clonal variants of *U. parvum* serovar 3 (27815^T^-K5, DR1-K1, M14-K16 and V892-K2) and from *U. urealyticum* serovars 9, 10 and 12 (Fig. 3). Although all clonal variants showed predominant expression of a specific protein, some also showed weak expression of truncated proteins or of proteins deriving from alternating expression. The clonal variant DR1-K1 showed a strong reaction of UU172, but also showed a faint band of the fusion protein UU171/144 (Fig. 3, lane 2). The clonal variant M14-K16 showed a strong band of the fusion protein UU171/144, but also expressed a truncated UU172 protein (Fig. 3, lane 3). The UU172 protein is truncated in this variant, due to a nonsense mutation at position 1132 in the UU172 ORF (Accession No. FN601250). Protein expression of the three *U. urealyticum* serovars 9, 10 and 12 was in agreement with their GenBank sequences; i.e. serovars 9 and 12 predominantly expressed UU172 and serovar 10 expressed UU171/144. A faint band was also observed in Western blots of total protein of serovar 10 when reacting with Pab α-U171 (Fig. 3, lane 6), which might be a truncated UU171/144 protein. Also, serovar 10 showed a faint reaction of UU172, indicating that alternate gene expression within the UU172 element likewise had occurred in this strain. Faint bands obtained with Pab α-U172C could also be observed in several strains/variants between 34 and 43 kDa. These bands appeared at the same molecular weight as the truncated UU172 protein of M14-K16, indicating that the mutation at this site might be a frequently occurring event. The clonal variant 27815^T^-K5 showed a positive signal only for the UU172 protein, while expression of the UU144 and UU171 proteins were not detected (Fig. 3, lane 1).

**Fig. 4:**
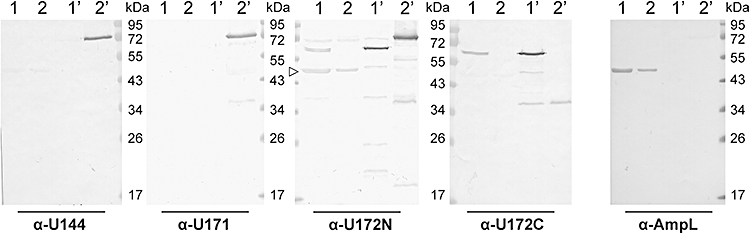
Western blot analyses of Triton X-114 phase partitioned total protein of *U. parvum* serovar 3 strains DR1 and M14. Lanes 1 and 2, hydrophilic fractions of DR1 and M14 and lanes 1′ and 2′, hydrophobic fractions of DR1 and M14 respectively. Proteins were detected with monospecific Pabs (see Table S3) indicated below the blots. α-AmpL is a monoclonal antibody against the cytosol aminopeptidase (UU507). Open triangle: background bands in the hydrophilic phases which were already detectable with pre-immune sera (Fig. S6).

To show that the two proteins UU172 and UU171/144 are potential membrane proteins, Triton X-114 phase partitioning was carried out with total protein from strains DR1 and M14. Both hydrophilic and hydrophobic fractions were investigated by Western blot analysis with the four Pabs (Fig. 4). To qualitatively test for the efficiency of the protein fractionation, one nitrocellulose membrane was treated with a monoclonal antibody directed against the cytosol aminopeptidase AmpL, encoded by UU507. Western blot analysis showed that both the UU172 protein of strain DR1 and the fusion protein UU171/144 of strain M14 are hydrophobic. A number of cleavage products or proteins of variable length could be observed in the hydrophobic fractions with Pabs α-U171, α-U172N and α-U172C. A double band of the UU172 protein could also be detected in the hydrophilic fraction of strain DR1 with Pabs α-U172C and α-U172N, probably as a result of incomplete fractionation. The majority of the proteins were, however, found in the hydrophobic fraction. The observed double band indicates that the protein had been processed and cleaved from its potential signal peptide.

### Phase variation in expression of the UU172 element during selective antibody pressure

**Fig. 5:**
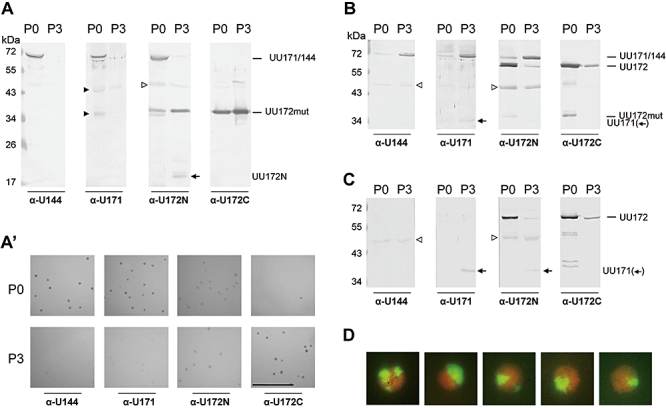
Phase variation of clonal variants of *U. parvum* serotype 3 before and after exposure to monospecific Pabs. A–C. Western blot analyses of: (A) M14-K16 before (P0) and after (P3) serial exposure to Pab α-U144, (B) DR1-K1 before and after serial exposure to Pab α-U172C, and (C) 27815^T^-K5 before and after exposure to Pab α-U172C. Proteins were detected with monospecific Pabs (see Table S3) indicated below the blots. Black triangles: cross-reaction with the UU376 protein. Open triangles: background bands, which were already detectable with pre-immune sera. A′. Colony immunoblots of M14-K16 before and after serial exposure to Pab α-UU144. Cells were plated before antibody treatment (P0) and after the third passage (P3) after antibody treatment. Scale bar: 1 mm. For immunostaining, monospecific Pabs (see Table S3) indicated below the blots were used. D. Colony immunoblots showing sectored colonies of subclonal populations of M14-K16, which had been exposed to Pab α-U144. Double immunofluorescence overlay: green (bright) sectors, UU171/144 expression and red (dark) sectors, UU172 expression.

To investigate whether the UU172 element is phase-variable within propagating populations, clonal variants of *U. parvum* serovar 3 were passaged three times in broth containing Pabs directed either against the C-terminal region of the UU172 protein (Pab α-U172C) or against the fusion protein UU171/144 (Pab α-U144). The clonal variants 27815^T^-K5 and DR1-K1 were exposed to Pab α-U172C and clonal variant M14-K16 was exposed to Pab α-U144. Expression of UU172 and UU171/144 was monitored by Western blot analysis and in colony blots. Both techniques revealed that the antibody treatment led to the emergence of escape variants expressing alternate proteins that had not been the targets of selective pressure. Selective pressure against the fusion protein UU171/144 in M14-K16 yielded clones predominantly expressing UU172 (Fig. 5A and A′) and selective pressure against UU172 yielded clones predominantly expressing the fusion protein UU171/144 in DR1-K1 (Fig. 5B) and UU171 in 27815^T^-K5 (Fig. 5C). In all cases, proteins were always expressed as fusions with the N-terminal part of UU172. A frequently observed variation was a truncated UU172 (UU172mut) protein or a truncated form of UU172 comprising only the N-terminal part of UU172 (UU172N). The latter could also be observed in the strains V890 and V397 (Fig. S3) (Accession No. FN601251, FN601252), where an insertion of an additional thymidine at the potential inversion sequence led to a nonsense mutation, resulting in a stop codon in the 3′ region of UU172N.

Antibody pressure with monospecific Pabs directed against the three *U. urealyticum* strains or against clonal lineages of these did not select for clones with alternating expression. However, colony and Western blots showed that altered gene expression likewise occurred in clonal variants of serovars 9 and 10, even without antibody pressure (not shown). Altered expression was observed in one to three cells per 100 cells in clonal variants of serovars 9 and 10; i.e. approximately 1% of the clones of serovar 9 expressed UU171/144 and approximately 1% of serovar 10 expressed UU172. Similar results were obtained for subclonal lineages deriving from clonal variants DR1-K1 and M14-K16. Reversion of phase variation was observed in subclonal populations of antibody-treated M14-K16 using the double immunofluorescence technique to immunostain colony blots, as shown in Fig. 5D by the sectored expression of UU171/144 in predominantly UU172-expressing colonies after exposure of cells to Pab α-U144.

### Site-specific DNA inversion within the UU172 element leads to alternate phase variation between UU172 and UU171

**Fig. 6:**
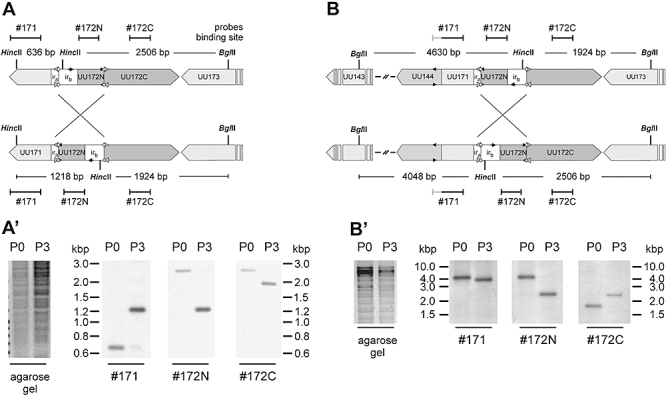
DNA inversion in the UU172 element of *U. parvum* serotype 3 clonal variants 27815^T^-K5 and M14-K16. Schematic illustration of the DNA inversion event in (A) 27815^T^-K5 and (B) M14-K16. Southern blot analyses of (A′) 27815^T^-K5 before (P0) and after (P3) selective pressure with Pab α-U172C and (B′) M14-K16 before and after selective pressure with Pab α-U144. Genomic DNA was digested with BglII and HincII and hybridized with the DIG-11-dUTP-labelled probes (see Table S3) indicated below the blots.

The configuration of the UU172 element, before and after antibody treatment, was characterized in the two clonal variants 27815^T^-K5 and M14-K16 by Southern blot analysis (Fig. 6). Genomic DNA was extracted from cells that had been cultivated at standard growth conditions and from cells that had been passaged three times in the presence of either Pab α-U172C or Pab α-U144. Genomic DNA was digested with the site-specific endonucleases BglII and HincII, and the orientation of the 5′ region of UU172 (UU172N) and the intergenic spacer region upstream of UU172 (ir_b_) was monitored by Southern blot hybridization of the DNA fragments with a probe directed against the UU172N sequence. Additionally, the orientations of the sequences encoding UU171 and the C-terminal part of UU172 were detected with probes targeting the UU171 and U172C sequences respectively.

In strain ATCC 27815^T^, the ORF UU171 has a HincII restriction site at its 3′ terminus, which is missing in the strains where the UU171 and UU144 sequences are fused. The choice of restriction sites allowed excision and partitioning of the UU172 element into two fragments. In both strains ATCC 27815 and M14, a HincII restriction site lies within the intergenic spacer region ir_b_, just behind one of the postulated inversion site (Fig. 6A and B). Inversion of the UU172N/ir_b_ region causes a relocation of the HincII site, leading to altered fragment lengths. Southern blot analysis showed that two locus configurations preferentially occurred in the two investigated variants; one with the UU172N/ir_b_ region in front of UU172C, and the other with this region inverted and located in front of UU171. The majority of the population of 27815^T^-K5 grown under standard conditions had the locus configuration where UU172N was fused with UU172C. Only a single band of the expected 2506 bp was detected with the probe against the UU172N sequence (Fig. 6A′). After antibody treatment, inversion of the UU172N/ir_b_ region resulted in a band of 1218 bp. The faint band of 636 bp observed in Fig. 6A′, when detected with probe #171, indicated that a minor population still contained the original locus configuration. By cloning of genomic DNA of 27815^T^-K5 after antibody pressure clones with an inverted sequence were obtained (Accession No. FN601248). The same inversion was observed in clonal variant DR1-K1 (Accession No. FN601254). In clonal variant M14-K16, inversion of the UU172N/ir_b_ region after treatment with Pab α-U144 resulted in a single band of 2506 bp in blots probed with #172N. Inversion of the suggested region was proven with all three probes (Fig. 6B′) and by sequencing of cloned genomic DNA (Accession No. FN601250).

### Site-specific DNA inversion of the UU172 element in *U. parvum* serovar 3 strain V892

**Fig. 7:**
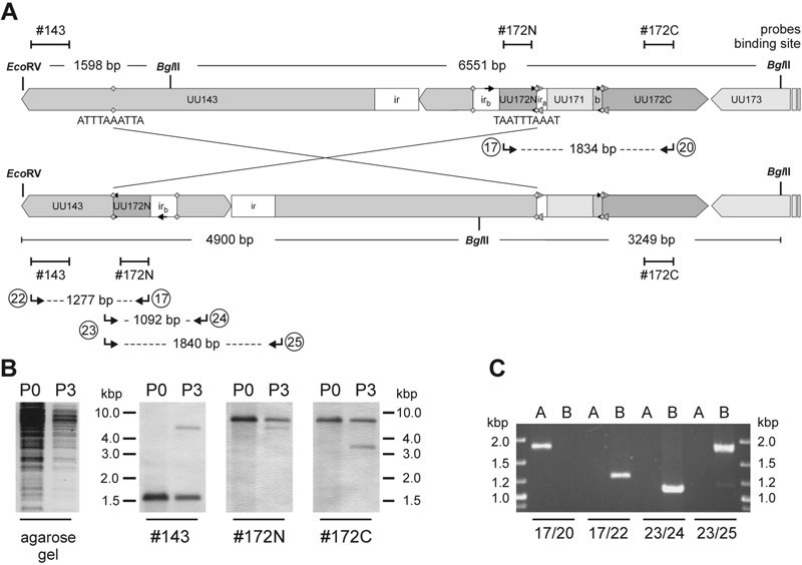
DNA inversion of the UU172 element in *U. parvum* serotype 3 clonal variant V892-K2. A and B. (A) Schematic illustration of the DNA inversion event and (B) Southern blot analysis of V892-K2 before (P0) and after (P3) selective pressure with Pab α-U172C. Genomic DNA was digested with BglII and EcoRV and hybridized with the DIG-11-dUTP-labelled probes (see Table S3) indicated below the blots. C. PCR analysis of subclones A and B with primer pairs 17/20, 17/22, 23/24 and 23/25 (see Table S3).

The organization of the UU172 element in clonal variant V892-K2 was unique among the observed serovar 3 strains, as it predominantly expressed a fusion protein that contained both UU172 and UU171 and a partial sequence of UU144 (Fig. 1). To investigate antigenic variation in this clonal variant, selective antibody pressure was directed against the C-terminal part of UU172 with Pab α-U172C. Populations before and after selective pressure were investigated by Southern blot analyses and by cloning and sequencing of genomic DNA of the UU172 element. The cloned genomic DNA showed that at least two variations of the UU172 element had evolved after selective antibody pressure; one where the locus arrangement was unaltered, but a mutation had appeared in the 5′ region of UU172C (Accession No. FN601256), and another where an inversion had occurred, in which part of the ORF UU143 was fused to UU172C (Accession No. FN601257) and UU172N was fused to the C-terminal part of UU143 (Fig. 7A). Southern blot analysis of EcoRV- and BglII-digested genomic DNA confirmed these results, detecting both the original and an altered locus configuration (Fig. 7B). BglII was chosen, as one restriction site lies outside the UU172 element in ORF UU173 and another in the ORF UU143 within the inverted fragment. The restriction site is relocated after inversion, which could be detected with the probes #143, #172N and #172C. Two subclones were isolated from the population after selective antibody pressure, each possessing one of the genomic arrangements described above. Fusion of the UU172N sequence with the C-terminal part of ORF UU143 in subclone B was shown by PCR analysis (Fig. 7C). Sequencing of the PCR product showed that the fusion between UU172N and UU143 was in frame. The same gene fusion could not be detected by PCR analysis in subclone A.

## Discussion

Phase switching of genes expressing surface membrane protein antigens is not uncommon in the genus *Mycoplasma* (Yogev *et al*., 2002; Citti *et al*., 2010). It is an important microbial strategy for host adaptation and evasion of immune responses to maintain diversity in a propagating population. Well-described examples of phase-variable loci include the *vsp* genes of *Mycoplasma bovis* (Lysnyansky *et al*., 2001a,b;), the *vsa* genes of *Mycoplasma pulmonis* (Bhugra and Dybvig, 1992; Bhugra *et al*., 1995) and the *vpma* genes of *Mycoplasma agalactiae* (Glew *et al*., 2000; 2002; Flitman-Tene *et al*., 2003; Chopra-Dewasthaly *et al*., 2008). The mechanism governing phase variations in these species is thought to be driven either by site-specific DNA inversions which link the ORF of a silent gene to a unique active promoter, as observed in the *vsp* genes of *M. bovis* and the *vpma* genes of *M. agalactiae* (Lysnyansky *et al*., 2001b; Glew *et al*., 2002; Flitman-Tene *et al*., 2003), or by juxtaposition of a DNA sequence containing promoter, ribosome binding site and 5′ terminal region of an ORF in front of the 3′ end of a previously silent gene, as reported for the *vsa* genes in *M. pulmonis* (Bhugra *et al*., 1995; Shen *et al*., 2000). The latter mechanism has recently been suggested to occur within the *mba* locus of *U. parvum* serovar 3 (Zimmerman *et al*., 2009) and we suggest that it also occurs in its UU172 element.

Based on sequence data from *U. parvum* serovar 3 strains and on the data provided by the J. Craig Venter Institute for all 14 serovars, we propose that the three ORFs designated UU144, UU171 and UU172 in *U. parvum* serovar 3 strain ATCC 700970 comprise a phase-variable element for expression of different surface exposed proteins. The ORFs UU144 and UU171 of *U. parvum* serovar 3 were both originally annotated as ‘conserved hypothetical’ encoding putative proteins with expected molecular weights of 27.8 kDa and 22.6 kDa respectively (Glass *et al*., 2000). Here, we provide evidence that neither ORF is expressed alone, but only in conjunction with the N-terminal sequence of UU172. As a result, these genes have been re-annotated in the genome sequences of ATCC 700970 and the ATCC type strains of all 14 known serovars.

The locus configuration of the two sequenced *U. parvum* serovar 3 strains differs from those of all investigated strains, in that it is fractionated by a 20 kb genomic region comprising 26 potential ORFs (UU145–UU170). In Fig. 1, we have divided the ORF UU144 of strain ATCC 700970 into three parts (a, b and c). Of these, part a, which comprises 14 amino acids, only occurs in the fully sequenced serovar 3 strains. We believe that, due to the disruption of the UU171/144 fusion in the ATCC 700970 strain, and also in the type strain ATCC 27815^T^, the 5′ end of UU144 was falsely annotated and the ORF is actually a pseudogene in these two strains. No band for the UU144 protein was detected by Western blot analysis of total protein of *U. parvum* serovar 3 strain ATCC 27815^T^, indicating that this ORF does not possess its own promoter and consequently is not expressed in this strain. Part b of UU144 comprises amino acids 15–35 (DYDVKLTNVKIANIALNNDNV) in *U. parvum*, and was located at a different site in variant V892-K2 (Fig. 1). The amino acids DYD are the linker between the ORFs UU171 and UU144 in *U. parvum* strains. Part c of UU144 comprises amino acids 36 up to the end, except in *U. parvum* serovar 6, where the potential ORF is truncated due to a mutation.

The disruption of the UU171/144 fusion seems untypical and could not be observed in any of the investigated serovar 3 strains. Rather, the ORF fusion UU171/144 seems to represent the prevalent genomic organization in both species. Interestingly, the genomic fragment that disrupts the ORF in the two sequenced serovar 3 strains is located near the potentially size- and phase-variable *mba* locus in *U. parvum* serovar 6 and in *U. urealyticum* serovars 5, 7, 8, 10, 11 and 12. In several other strains, this genomic region seems to be missing altogether, indicating relocation or deletion of this region during evolution. With microarray-based genome analysis, Momynaliev *et al*. demonstrated that this region can be present or absent in serovars 1, 3 and 6 (Momynaliev *et al*., 2007). They suggested that, due to its genetic features, this region could be a pathogenicity island deriving from foreign DNA. Interestingly, an *in silico*-based deletion of the 20 kb sequence between two direct repeats (ATAATCA) in the ORFs UU144 and UU171 of strain ATCC 700970 results in the UU172 locus organization shown in Fig. 1A. In *U. urealyticum* serovar 10, the 20 kb region is flanked by 22 bp (TAATCGTGATTATTGAACCTTG) direct repeats. This, and the fact that several potential integrase/recombinase genes (e.g. UU145, a close homologue of *rip*X and UU154, a close homologue of phage recombinase Bet) are located on the 20 kb region, raise the questions if it is a mobile element and if the direct repeats promote excision of the region.

By sequencing and through blast analyses, we could identify at least six different locus configurations (Figs 1 and 7), and we suggest that the two major configurations depicted in Fig. 1A and B are phase-variable at high frequency in both *U. parvum* and *U. urealyticum*. Phase variation of UU171 and UU172 expression were previously suggested to occur as a result of slipped-strand mispairing at a poly-AT (AT9) tract located in the intergenic region between the ORFs (Rocha and Blanchard, 2002). Here we provide evidence that phase variation occurs at two inverted repeat regions, of which one is located in the ORF UU172 and another in the intergenic spacer region between UU172 and UU171. Three 21-nt-long inverted repeat regions, which were proposed for the ON/OFF switching of the *mba* gene, were localized in the *mba* locus (Zimmerman *et al*., 2009). Similar inverted repeats, but with a different assembly, were found in the UU172 element. The UU172 ORF contains a 51-nucleotide-long sequence (TTAAATAATGATAATTTAAATTATCAAACAGTAACTTTTGAACAAGTTCCT) encoding LNNDNLNYQTVTFEQVP, of which one part (TTAAATAATGATAAT) is also located in UU144 and another part (TTAAATTATCAAACAGTAACTTTTGAACAAGTTCCT) on the complementary strand of the intergenic spacer region between UU172 and UU171 (Fig. 2). Inversion seems to take place at all three regions, but seems to be most frequent between the two long inverted repeats. Inversion frequencies between UU172 and UU171/144 expression were measured in clonal variants with the monospecific Pabs α-U144 and α-U172 (Fig. S4). Approximately 1–2% of the populations were found to spontaneously switch their expression to the opposite side without the application of selective antibody pressure. Switching was also observed as sectoring in colony blots (Fig. 4D), indicating that phase variation in expression occurs at high frequency. This result is in agreement with previous results obtained for the *mba* locus (Monecke *et al*., 2003) and indicates that phase variation of the UU172 element must be a mechanical event processed by specific enzymes. Determining the exact switching frequency is, however, hampered by sectoring of the colonies and by point mutations within the locus, such as the occasionally appearing mutation in the 3′ region of UU172N, which leads to false-negative detection.

Both orientations of the UU172 element encode potential membrane proteins, which are immunogenic in rabbits. Hyperimmune antisera, which were generated against whole-cell preparations of the two isolates DR1 and M14, reacted with the recombinant proteins, and the reaction patterns was in agreement with the expression profiles of the strains (Fig. S5), i.e. antiserum against DR1 reacted strongly with U172N and U172C and the antiserum against M14 reacted strongly with U144, U171 and UU172N. Additional work is, however, necessary to prove that both potential membrane proteins are indeed immunogenic in the human host and that phase variation of the UU172 element, as a consequence of immune evasion, similarly occurs *in vivo*.

Size variation of proteins encoded by the UU172 element is achieved by point mutations within its ORFs. A frequently observed mutation was an insertion of a thymidine in the 3′ region of the inverted repeat. This mutation causes a translational frameshift in the ORF, which results in a truncated protein comprising only the N-terminal part of UU172 (UU172N). Such mutations are not infrequent among mycoplasmas and were described to occur within short homopolymeric tracts of adenine residues (poly-A region) located in the N-terminal encoding region of the *vaa* gene of *Mycoplasma hominis* (Zhang and Wise, 1997; 2001;). The genomic sequence of *U. parvum* serovar 6 also shows an alteration in another poly-A region in the UU171/144 sequence, leading to a potentially truncated protein upon *in silico* inversion of the database sequence.

Other mutations observed that caused potential size variation of proteins were the nonsense mutations G1,132T (GAA→TAA) in clonal variant M14-K16 (Accession No. FN601249) and G490T (GAA→TAA) in subclones of V892-K2, which had been exposed to Pab α-U172C (Accession No. FN601256). Bands with equal molecular weight to the one observed in M14-K16 were also seen in Pab α-U172C-immunostained Western blots of clonal populations of other strains, indicating that this mutation occurs frequently. Since we never detected a complete UU172 protein in the strain M14, we presume that the mutation is either irreversible or reversion occurs at very low frequency. Such a low frequency or irreversible nonsense mutation, in which guanine is replaced with the nucleotide thymidine, has been reported for the *pvp*A gene of *Mycoplasma gallisepticum*, where the mutation takes place consistently in the fourth GAA codon of a poly-GAA tract (Boguslavsky *et al*., 2000).

**Fig. 8:**
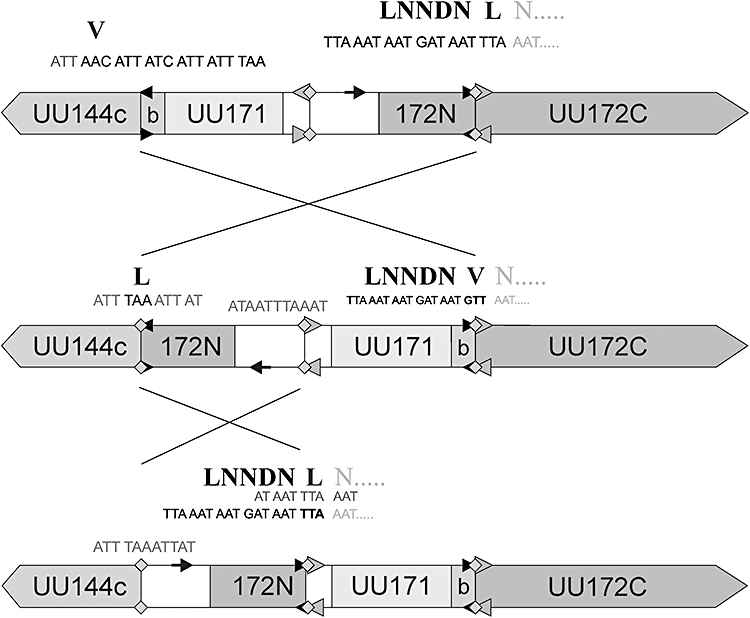
Schematic illustration of possible DNA inversion events leading to the UU172 element observed in strain V892. In a first inversion event, part ‘b’ of the ORF UU144 was fused, in frame, to UU172C and UU172N was fused to part ‘c’ of UU144. In a second inversion event at the inverted repeat ATTTAAATTAT, UU172N was joined, in frame, with the intergenic region in front of UU171, thereby creating a long ORF comprising UU172N, UU171, part of UU144 and UU172C.

Among the *U. parvum* serovar 3 strains and clonal isolates, the clonal variant V892-K2 possesses an exceptional UU172 locus organization. Its chimeric gene arrangement might yield valuable information about a possible core sequence in inversion sites located throughout the genome. In this clonal variant, selective antibody pressure led to an inversion at a completely different site than expected. In subpopulations of this clonal variant, the sequence encoding UU172N was fused to the C-terminal part of the ORF UU143, suggesting that the UU172 element might expand even beyond the ORFs described in this report. The new potential chimeric gene was in frame at the inversion site. Inversion had taken place at a site with the nucleotide sequence ATTTAAATTA. This short homologous sequence appears within the long inverted repeats mentioned above and it occurs in multiple copies within the UU172 element and the UU143 ORF. A similar sequence can be located in the suggested inversion sites of the *mba* locus, where the sequence reads ATTTGAATTA. If this short sequence would serve as a core sequence for DNA inversion, the arrangement of the UU172 element in V892-K2 could be explained as the result of the two inversions depicted in Fig. 8, although these inversion events could not be proven by PCR and no genomes with the first two locus organizations were obtained by subcloning. Short inverted repeats of only 14 and even 9 bp, promoting site-specific DNA inversion, have been suggested for flagellar phase variation in *Salmonella* and for phase variation of the type 1 pili in *E. coli* (Zieg and Simon, 1980; Abraham *et al*., 1985). In *Mycoplasma penetrans*, inverted repeats of only 12 bp serve as inversion sequences in the promoter regions of the *mlp* genes (Horino *et al*., 2009). An alignment of inverted repeat regions of 30 *mlp* genes showed that they all differed from each other, suggesting that the active recombinase MYPE2900 has flexibility in recognizing the recombination sites (Horino *et al*., 2009). A similar flexible mechanism could likewise exist in ureaplasmas.

All three sequenced *U. urealyticum* strains ATCC 27814 (serovar 2), ATCC 27816 (serovar 4) and ATCC 33696 (serovar 12) have the same novel UU172 element organization that deviates from the locus arrangement depicted in Fig. 1A. Although this ‘original’ locus organization can be obtained by *in silico* inversion at two sites, one within UU144 and the other in the intergenic spacer region between UU172 and UU171, it is not clear if this inversion had occurred as an error and if it is reversible. Selective antibody pressure against the UU172 protein of strain ATCC 33696 did not select for subclones with alternating expression.

The questions remain which enzymes are responsible for the DNA inversion and what is the core sequence recognized by potential recombinases. In several mycoplasma species, site-specific recombinases, exhibiting high homology to the tyrosine site-specific recombinase family proteins XerC/D of *E. coli* and RipX or CodV of *Bacillus subtilis*, were found to be responsible for the DNA inversions within the phase-variable loci (Ron *et al*., 2002; Sitaraman *et al*., 2002; Chopra-Dewasthaly *et al*., 2008). For *U. parvum* serovar 3, three such recombinases were annotated (Glass *et al*., 2000), of which two, XerC (UU222) and RipX (UU145), also occur in *U. urealyticum* serovar 10. Current studies are in progress to investigate the involvement of these recombinases in the proposed DNA inversions.

Together with the *mba* locus, ureaplasmas possess at least two sets of genes that use chromosomal inversion to alter their surface antigen expression. Since infections with either *Ureaplasma* species are in most cases asymptomatic, an intriguing question to be resolved is to elucidate the role that antigenic variation plays in the host immune response and how antigenic variation correlates with the ureaplasma pathogenicity. It has been reported that the Triton X-114 detergent phase of *U. parvum* can generate an inflammatory response by inducing the nuclear factor NF-κB through the Toll-like receptor TLR2 (Shimizu *et al*., 2008). Since the MBA was only one of several components responsible for this inflammatory response, we speculate that recognition of both antigenic variants could be a cause of ureaplasma disease. In this scenario, patients who only recognize the dominant form of MBA and UU172 would only develop a minor inflammatory response without any symptoms. Phase variation in both the *mba* locus and the UU172 element could then elevate the inflammatory response. Alternatively, expression of the fusion protein UU171/144 or loss of expression due to mutations could camouflage the organism so that it would be capable of evading the immune response and execute its host-damaging effect via its virulence factors. Interestingly, only one of the five patient isolates analysed in this study showed predominant expression of UU172. Also, most of the 14 ATCC type strains express the same ∼500-amino-acid version of UU172, which could be the consequence of prolonged laboratory passaging. Knowing how ureaplasmas change their antigenic make-up could be critical in understanding why a minority of patients develop clinical symptoms like non-gonococcal urethritis or a variety of adverse pregnancy outcomes.

## Experimental procedures

### *Ureaplasma* clonal variants and cultivation

Six clonal variants of *U. parvum* serovar 3 strains were used: a clonal variant of type strain ATCC 27815^T^ (27815^T^-K5) and five clonal variants (Kn) from clinical isolates DR1, M14, V892, V890 and V397 (DR1-K1, M14-K16, V892-K2, V890-K12 and V397-K18 respectively). Clonal variants derived from single colonies of the corresponding strain. Additionally, the following *U. urealyticum* strains were used: serovar 9 (strain ATCC 33175), serovar 10 (strain Western CX3) and serovar 12 (strain JSL-U24-CX3) (MacKenzie *et al*., 1996). *Ureaplasma* strains and clonal variants were cultivated in ureaplasma medium (UPM) as previously described (Zimmerman *et al*., 2009).

### Recombinant proteins

Four recombinant proteins were expressed as fusion proteins with the maltose-binding protein using the expression vector pMAL™-c2X and purified by affinity chromatography as described by the manufacturer (New England BioLabs). Briefly, DNA was extracted as described (Dumke *et al*., 2003) from *U. parvum* type strain ATCC 27815^T^ and used as template for PCR. PCR products were amplified with *Pfu* Polymerase (Fermentas Life Science) and primers (Table S3) containing specific endonuclease cleavage sites (5′ BamHI and 3′ HindIII or SalI) and designed for in-frame fusion with the *mal*E gene. To eliminate the three UGA codons in construct U172C, four PCR products were synthesized and fused by either blunt end or NheI site-specific ligation. All cloned products were sequenced (GATC-Biotech, Germany) to confirm their identity.

### Monospecific polyclonal antibodies (Pabs)

Monospecific hyperimmune antisera were generated in rabbits against each of the recombinant proteins (Table S3) using a protocol previously described (Ruhnke and Rosendal, 1994).

### Serial transfer in the presence of monospecific Pabs

*Ureaplasma* clonal variants were passaged three times in UPM containing either α-U172C or α-U144. Prior to addition to media, sera were incubated at 56°C for 30 min to inactivate complement components. Ureaplasma cultures containing 10^3^–10^4^ colony-forming units (cfu) per ml were incubated for 24 h at 37°C with Pabs at dilutions of 1:10 or 1:100. The next-day cultures were diluted to contain 10^3^–10^4^ cfu ml^−1^ and were passaged twice more under the same conditions.

For Western blot analysis, cells before antibody treatment (P0) and from the third antibody-treated passage (P3) were incubated in 500 ml of UPM at 37°C for 24 h without the selective pressure of Pabs. Cells were centrifuged and washed as previously described (Monecke *et al*., 2003).

To obtain colonies for preparing colony immunoblots, cells before antibody treatment and from the third antibody-treated passage were diluted and incubated on UPM agar for 48 h at 37°C and 5% CO_2_.

### Immunostaining of colony blots by double immunofluorescence

Double immunofluorescence of colony blots was carried out as described (Duffy *et al*., 1998), except that 1× Roti®-Block (CARL ROTH) was used as blocking agent in 1× TBS. Subclonal populations of clonal variant M14-K16, which had been exposed to α-U144, were incubated on UPM agar and transferred onto nitrocellulose. As primary antibody, to detect UU171/144-expressing cells, α-U144 (1:500) was used and labelled with a Texas red-conjugated α-rabbit IgG (Acris Antibodies) (1:1 000) as secondary antibody. The protocol was repeated with α-U172C (1:500) as primary and a FITC-conjugated α-rabbit IgG (Acris Antibodies) (1:1000) as secondary antibody to counterstain the colonies. Images of colonies were made with an Olympus AX70 fluorescence microscope and a 20× objective with the fluorescence imaging software cell∧F. Colours were reversed for better illustration.

### Triton X-114 phase partitioning

Triton X-114 phase partitioning was carried out as described (Kiarie *et al*., 1996) with cells from 1 l of ureaplasma culture, except that proteins were methanol/chloroform-precipitated. Proteins from the hydrophilic phases were resuspended in 200 µl and from hydrophobic phases in 100 µl of sample buffer. For SDS-PAGE analysis, proteins of 20 µl of samples per run were separated in 12.5% gels.

### Southern blot analyses

Genomic DNA was isolated as described (Dumke *et al*., 2003) from 500 ml of *U. parvum* serotype 3 cultures. DNA pellets were air-dried and resuspended in 100 µl of H_2_O for digestion with site-specific restriction endonucleases. Genomic DNA derived from DR1-K1 and M14-K16 was digested with 50 U of BglII (Fermentas Life Science) and 50 U of HincII (New England BioLabs) overnight at 37°C. Genomic DNA deriving from V892-K2 was digested with 50 U of BglII (Fermentas Life Sciences) and 50 U of EcoRV (Fermentas Life Sciences). The digested DNA (20 µl lane^−1^) was separated in a 1% agarose gel and transferred to nylon membranes (Sambrook *et al*., 1989).

Four DIG-11-dUTP (Roche Applied Science)-labelled PCR products were synthesized for use as hybridization probes (Table S3): one from UU171 (#171), one from the corresponding N-terminal sequence of UU172 (#172N), one from the corresponding C-terminal part of UU172 (#172C) and one from the corresponding C-terminal sequence of ORF UU143 (#143). Hybridization and detection were carried out as described (Chopra-Dewasthaly *et al*., 2005).

### Sequencing of the UU172 element

Genomic DNA was isolated and digested with BglII as described above. The digested DNA was ligated into the unique BamHI site of the vector pBC (Stratagene), and *E. coli* strain DH10B was transformed with the plasmids using the heat shock method. Positive clones were screened in colony blots with the DIG-11-dUTP-labelled PCR probes #171 or #172C (see Table S3). Plasmid inserts were sequenced by GATC-Biotech (Konstanz, Germany) and sequences were submitted to the DDBJ/EMBL/GenBank databases under the Accession No. FN601248, FN601249, FN601250, FN601251, FN601252, FN601253, FN601254, FN601255, FN601256 and FN601257.
