# Supplementary material for: Ureaplasma antigenic variation beyond MBA phase variation: DNA inversions generating chimeric structures and switching in expression of the MBA N-terminal paralogue UU172
Source: Mol Microbiol. 2011 Feb;79(3):663–76. doi: 10.1111/j.1365-2958.2010.07474.x (PMC3047005; doi:10.1111/j.1365-2958.2010.07474.x)
Supplement: Supplementary file 1 [file mmi0079-0663-SD1.pdf]

Species	serovar	strain [ATCC]	[U172]	locus tag	GenBank	NCBI reference Sequence	Description
Ureaplasma parvum	1	27813	x	UPA1_D0001	EDT48708.1	ZP_02691484.2	putative lipoprotein
Ureaplasma parvum	1	27813	xxx	UPA1_G0402	EDT49018.1	ZP_02931659.1	MBA N-terminal paralog of phase variable lipoprotein
Ureaplasma urealyticum	2	27814	xxx	UUR2_0166	EEH02482.1	ZP_03771915.1	conserved hypothetical protein
Ureaplasma parvum	3	700970	xxx	UU172	AAF30579.1	NP_078004.1	MBA N-terminal paralog of phase variable lipoprotein
Ureaplasma urealyticum	3	27815	xxx	UPA3_0179	ACA33003.1	YP_001752254.1	MBA N-terminal paralog of phase variable lipoprotein
Ureaplasma parvum	3	27815		uu172C	CBI70477.1		putative lipoprotein
Ureaplasma parvum	3	M14	xxx	uu172	CBI70480.1		MBA N-terminal paralog of phase variable lipoprotein
Ureaplasma parvum	3	V397		uu172C	CBI70484.1		putative lipoprotein
Ureaplasma parvum	3	DR1	xxx	uu172	CBI70486.1		MBA N-terminal paralog of phase variable lipoprotein
Ureaplasma parvum	3	V892	xxx	uu172N_171_144b_172C	CBI70488.1		MBA N-terminal paralog of phase variable lipoprotein
Ureaplasma parvum	3	V892	x	uu172N	CBI70489.1		MBA N-terminal paralog of phase variable lipoprotein
Ureaplasma parvum	3	V892		uu172C	CBI70490.1		putative lipoprotein
Ureaplasma urealyticum	4	27816	xxx	UUR4_0061	EDY74622.1	ZP_03205858.1	putative lipoprotein
Ureaplasma urealyticum	5	27817	xxx	UUR5_G0187	EDU06542.1	ZP_02964637.1	putative lipoprotein
Ureaplasma parvum	6	27818	xxx	UPA6_A0161	EDU19008.1	ZP_02971599.1	putative lipoprotein
Ureaplasma urealyticum	7	27819		UUR7_0167	EDU57041	ZP_02997003.1	conserved hypothetical protein
Ureaplasma urealyticum	8	27618	xxx	UUR8_0167	EEH01688.1	ZP_03772454.1	conserved hypothetical protein
Ureaplasma urealyticum	9	33175	xxx	UUR9_0558	EDX53885.1	ZP_03079775.1	putative lipoprotein
Ureaplasma urealyticum	10	33699		UUR10_0163	ACI59953.1	YP_002284568.1	hypothetical protein
Ureaplasma urealyticum	11	33695	xxx	UUR11_0162	EDU67029.1	ZP_03004006.1	putative lipoprotein
Ureaplasma urealyticum	12	33696	xxx	UUR12_A0163	EDX53110.1	ZP_03004175.1	putative lipoprotein
Ureaplasma urealyticum	13	33698		UUR13_0347	EDT49654	ZP_02931879.1	Part of the UU172-UU171-UU144 phase variable element. This is similar to the carboxyl terminus of UU172; however the amino terminus of gene in this serovar has iverted and is now linked to the UU171 ortholog.
Ureaplasma parvum	14	33697	xxx	UPA14_G0051	EDT87500.1	ZP_02958170.1	putative lipoprotein

x: only U172N
xxx: full length with U172N

**Species**	**serovar**	**strain [ATCC]**	**[U171/144]**	**locus tag**	**GenBank**	**NCBI reference Sequence**	**Description**
Ureaplasma parvum	1	27813		UPA1_G0401	EDT49176.1	ZP_02691092.1	conserved hypothetical protein
Ureaplasma parvum	3	M14	xxx	uu172N_171_144	CBI70478.1		MBA N-terminal paralog of phase variable lipoprotein
Ureaplasma parvum	3	V890	x	uu172N	CBI70482.1		MBA N-terminal paralog of phase variable lipoprotein
Ureaplasma parvum	3	V397	x	uu172N	CBI70483.1		MBA N-terminal paralog of phase variable lipoprotein
Ureaplasma parvum	3	DR1	xxx	uu172N_171_144	CBI70487.1		MBA N-terminal paralog of phase variable lipoprotein
Ureaplasma urealyticum	5	27817		UUR5_G0186	EDU06485.1	ZP_02554791.1	putative lipoprotein
Ureaplasma parvum	6	27818		UPA6_A0160	EDU18921.1	ZP_02554392.1	conserved hypothetical protein
Ureaplasma urealyticum	7	27819	xxx	UUR7_0166	EDU56898.1	ZP_02570029.2	putative lipoprotein
Ureaplasma urealyticum	8	27618		UUR8_0166	EEH01821.1	ZP_03772587.1	conserved hypothetical protein
Ureaplasma urealyticum	9	33175		UUR9_0559	EDX53889.1	ZP_03079779.1	conserved hypothetical protein
Ureaplasma urealyticum	10	33699	xxx	UUR10_0162	ACI60016.1	YP_002284567.1	multiple banded antigen
Ureaplasma urealyticum	11	33695		UUR11_0161	EDU66978.1	ZP_02556791.1	putative lipoprotein
Ureaplasma urealyticum	13	33698	xxx	UUR13_0348	EDT49608.1	ZP_02695322.2	putative lipoprotein
Ureaplasma parvum	14	33697		UPA14_G0050	EDT87644.1	ZP_02689934.1	conserved hypothetical protein

x: only U172N
xxx: full length with U172N

**Species**	**serovar**	**strain [ATCC]**	**[U171]**	**Locus tag**	**GenBank**	**NCBI reference Sequence**	**Description**
Ureaplasma parvum	1	27813		UPA1_D0002	EDT48707.1	ZP_02691485.1	conserved hypothetical protein
Ureaplasma parvum	3	700970		UU171	AAF30578.1	NP_078003.1	lipoprotein pseudogene
Ureaplasma parvum	3	27815		UPA3_0178	ACA33100.1	YP_001752253.1	lipoprotein pseudogene
Ureaplasma parvum	3	27815	xxx	uu172N_171	CBI70476.1		MBA N-terminal paralog of phase variable lipoprotein

xxx: full length with U172N

Species	serovar	strain [ATCC]	[U144]	Locus tag	GenBank	NCBI reference Sequence	Description
Ureaplasma urealyticum	2	27814		UUR2_0163	EEH02080.1	ZP_03771513.1	conserved hypothetical protein
Ureaplasma parvum	3	700970		UU144	AAF30550.1	NP_077975.1	Lipoprotein pseudogene
Ureaplasma parvum	3	27815		UPA3_0152	ACA33189.1	YP_001752227.1	Lipoprotein pseudogene
Ureaplasma parvum	3	M14		uu144	CBI70479.1		putative lipoprotein
Ureaplasma parvum	3	V890		uu144	CBI70481.1		putative lipoprotein
Ureaplasma parvum	3	DR1		uu144	CBI70485.1		putative lipoprotein
Ureaplasma urealyticum	4	27816		UUR4_0064	EDY74691.1	ZP_03205927.1	conserved hypothetical protein
Ureaplasma urealyticum	12	33696		UUR12_A0160	EDX53304.1	ZP_02557340.1	conserved domain protein

[U172]: orthologs of UU172 of *U. parvum* serovar 3 (ATCC 700970)
[U171]: orthologs of UU171 of *U. parvum* serovar 3 (ATCC 700970)
[U144]: orthologs of UU144 of *U. parvum* serovar 3 (ATCC 700970)
[U171/144]: : orthologs of UUR10_0162 of *U. urealyticum* serovar 10 (ATCC 33699)
pink: this study

Table S2 List of major ORFs appearing in the UU172 element of all 14 *Ureaplasma* serovars.

Primer no.	and designation	Primer sequence[a]	Localization 5'- 3'	Amino acids of ORF	Fusion protein	Pab[b]
1	U144f	TT**GGATCC**ATTGAACAAAATCAT	188918 – 188904	38 – 243	U144	α-U144
2	U144r	GG**AAGCTT**ATTTTTTATTTACTAC	188298 -–188315			
3	U171f	TT**GGATCC**GTGATTGCAAATAAAATA	210003 – 209986	1 – 175	U171	α-U171
4	U171r	TCG**AAGCTT**AATCAATTTCTAAACCAT	209475 – 209492			
5	U172Nf	TC**GGATCC**TCAAATACAAATATTAAATCT	210468 - 210488	31 – 132	U172N	α-U172N
6	U172Nr	CG**AAGCTT**AAACTAATGCTTCTGTTTTTA	210774 – 210754			
7	U172Cf1	TC**GGATCC**GATCAAAAATTAGAACCA	210825 – 210842	150 – 499	U172C	α-U172C
8	U172CrUGG2	TCT**GCTAGC**AA***CCA***ACGTAATCCTAAAAT	211126 – 211098			
9	U172Cf2	TTAGGATTACGTTGATT**GCTAGC**	211101 – 211123			
10	U172Cr2	ATTGTATAAATTAAATGTTGC	211388 – 211368			
11	U172CfUGG3	GGCTTTGAC***TGG***TCAACATTATTA	211389 – 211412			
12	U172Cr3	ATATTCAGCAATTTCTAAACGC	211547 – 211526			
13	U172CfUGG4	CCAAAAGAT***TGG***AACACTAGCAA	211548 – 211570			
14	U172Cr4	CGC**GTCGAC**TATTTTTTATTATTTG	211876 – 211861			
				**Probe name**		
15	#171f	TGCTATTAGTGCTACTGAA	209860 – 209860	#171		
16	#171r	CTACCCGGGAATTGCTAGAAACCA	209407 – 209421			
17	#172Nf	GCAATGTCATTAAGTGCGAT	210408 – 210427	#171N		
18	#172Nr	TTAAAACTAATGCTTCTGTT	210777 – 210758			
19	#172Cf	TACAACAATCATGAAAGCAA	211178 – 211197	#172C		
20	#172Cr	GCATTACTACAGGATCTTCA	211527 – 211508			
21	#143f	ACAAGATATGTTAGAAGCAAC	184510 – 184490	#143		
22	#143r	TGTTGGATAATCGAACTATTG	184092 – 184112			
23	#143r_3'	TTCATTTCCATCATCATCGTAGA	184949 – 184971			
24	#144r	AGCCATATGATACTTAGCAT	188559 – 188578			
25	#143r_5'	TACTCCACCGAATTTTCATCATT	187811 – 187833			

Table S3. Primers and binding sites within the ORFs UU172, UU171, UU144 and UU143 of *U. parvum* serovar 3 strain ATCC 700970 used for synthesizing recombinant fusion proteins for generating polyclonal antibodies and for probe synthesis.

[a]Nucleotides in bold represent restriction sites *Bam*HI , *Hind*III, *Nhe*I or *Sal*I. Nucleotides in bold, italics and underlined represent TGA to TGG modifications.

[b]Pab, monospecific polyclonal antibody used for detection

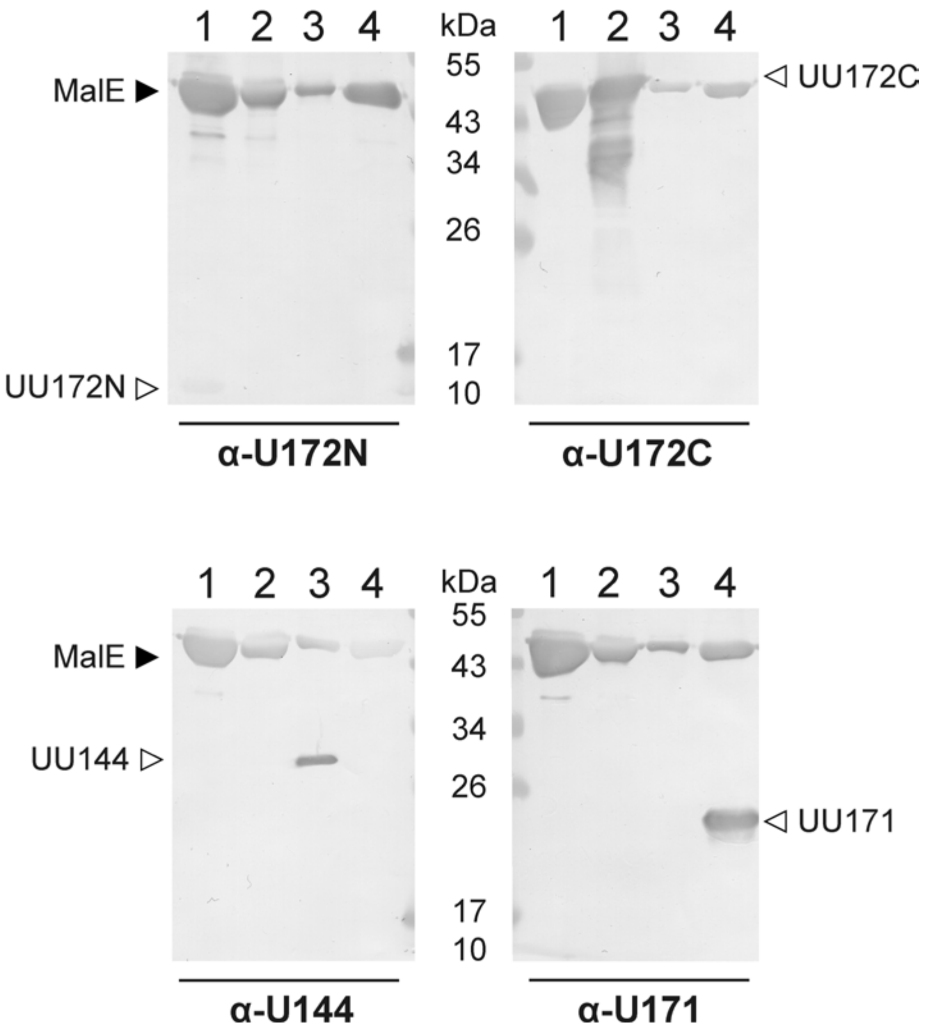

Fig. S1 Western blot analyses of Factor Xa-treated MalE fusion-proteins.

Maltose binding protein (MalE) fusion-proteins (MalE-UU172N, MalE-UU172C, MalE-UU144 and MalE-UU171) were purified by affinity chromatography as described by the manufacturer (NewEnglandBiolabs). Fusion proteins were digested with Factor Xa and detected with the monospecific polyclonal antibodies (see **Table S3**) indicated below the blots. Lane 1, MalE-UU172N, lane 2, MalE-UU172C, lane 3, MalE-UU144 and lane 4, MalE-UU171.

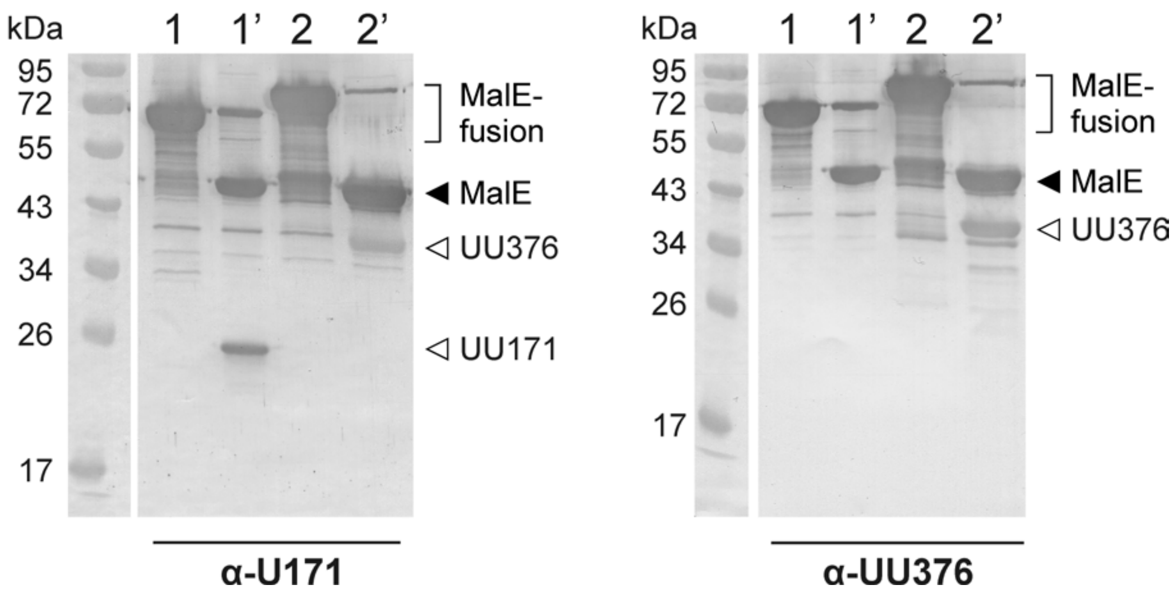

Fig. S2 Cross-reaction of α-UU171 with the UU376 protein.

Maltose binding protein (MalE) fusion-proteins (MalE-UU171 and MalE-UU376) were purified by affinity chromatography as described by the manufacturer (NewEnglandBiolabs). 2 µg / lane was loaded with undigested (lanes 1 and 2) and Factor Xa treated (lanes 1' and 2') protein. Lanes 1 and 1', MalE-UU171 and lanes 2 and 2', MalE-UU376. Proteins were detected with the monospecific antisera indicated below the blots.

Reference for MalE-UU376 and α-UU376: "Zimmerman, C. U., Stiedl, T., Rosengarten, R. and Spergser, J. (2009) Alternate phase variation in expression of two major surface membrane proteins (MBA and UU376) of Ureaplasma parvum serovar 3. FEMS Microbiol Lett 292: 187-193."

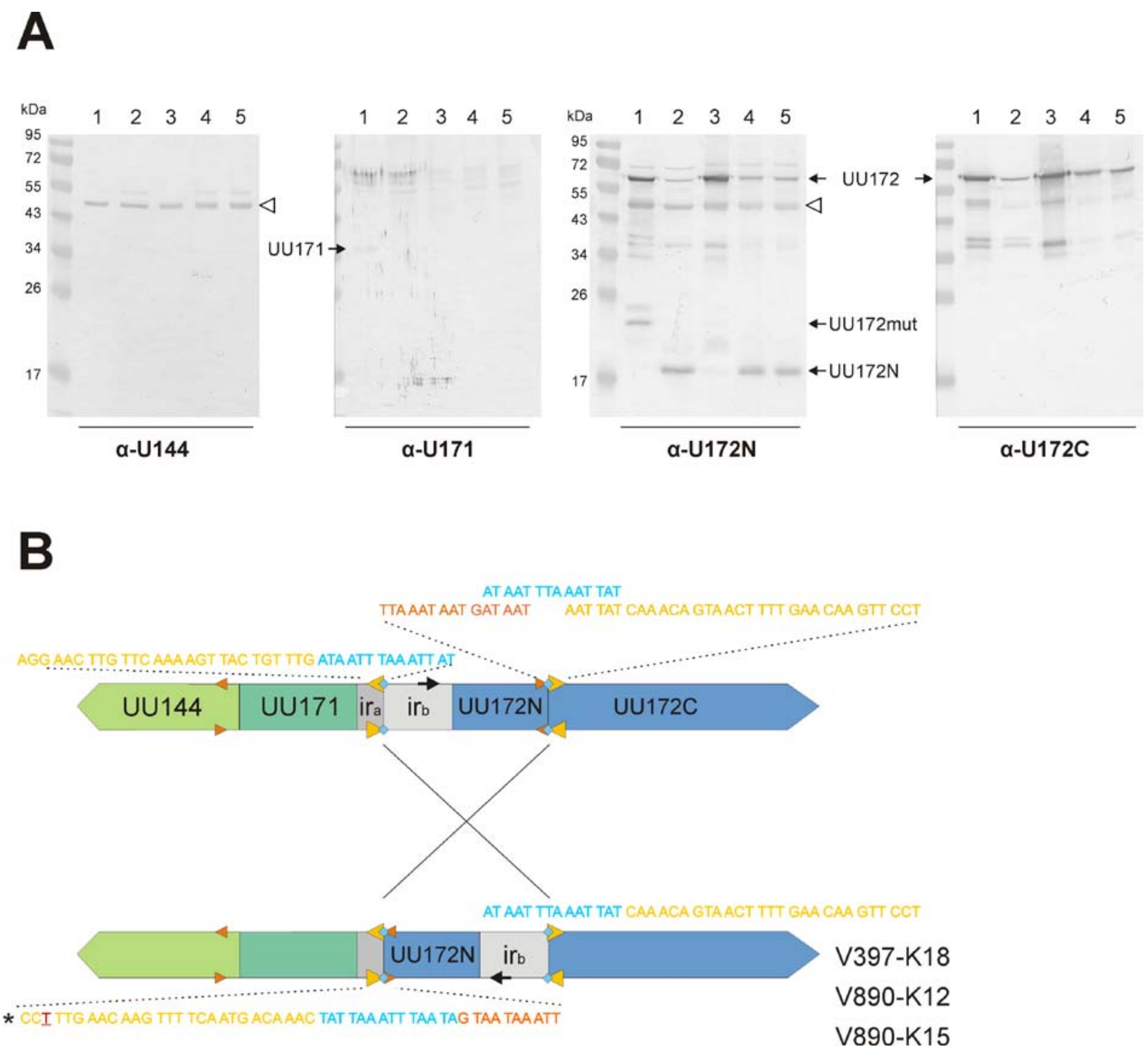

Fig. S3 Western blot analyses of total *Ureaplasma* cell protein and schematic illustration of DNA inversion. **A.** Western blot analysis with total cell protein of *U. parvum* serovar 3 clonal variants: lane 1, 27815^{T}-K5; lane 2, V397-K18; lane 3, V890-K2; lane 4, V890-K12; lane 5, V890-K15. Proteins were detected with monospecific Pabs (see **Table S3**) indicated below the blots. Open triangle: background bands in the hydrophilic phases which were already detectable with pre-immune sera (**Fig. S6**). **B.** Schematic illustration of DNA inversion in the UU172 element of clonal variants V397-K18, V890-K12 and K15, which lead to the expression of UU172N. Nucleotide in red and underlined: insertion of an additional thymidine leading to a nonsense mutation and resulting in a stop codon (*) in the 3' region of UU172N. Black arrow: potential promoter of the UU172 gene locus. ir_a and ir_b: intergenic spacer regions. Triangles and diamonds represent inverted repeat regions, where the diamond indicates a palindromic sequence (sequence in blue).

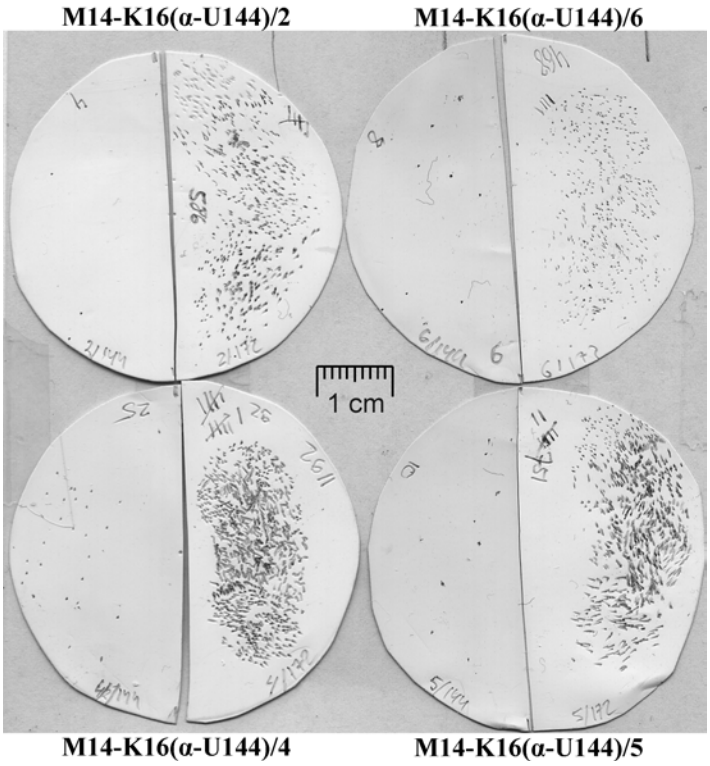

Fig S4 Detection of UU171/144 expressing revertants by colony immuno blot.

Over night cultures of clonal lineages (clones 2, 4, 5, 6) from the third passage of α-U144 treated M14-K14 were diluted and plated onto UPM agar. Colony lifts were detected with α-U144 and α-U172C and α-rabbit IgG (horseradish peroxidase conjugated). Colonies were marked with pencil.

Clonal variant	Number of Colonies detected with α-U144	Number of Colonies detected with α-U172C	Total positive	revertants [%]
M14-K16 (α-U144)/2	4	586	590	0.7
M14-K16 (α-U144)/4	25	1,192	1,217	2
M14-K16 (α-U144)/5	10	751	761	1.3
M14-K16 (α-U144)/6	6	468	474	1.2

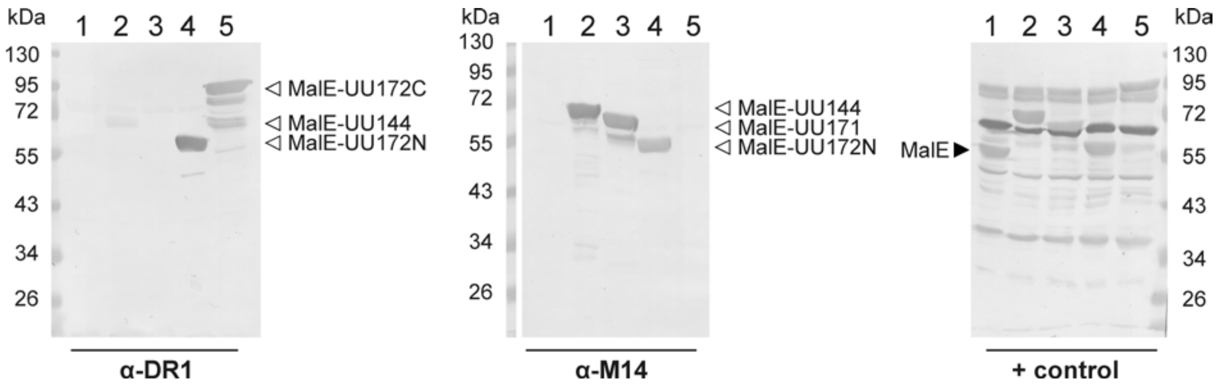

Fig. S5 Western blot with total protein from *E. coli* clones and anti-*Ureaplasma* hyperimmune antisera.

E. coli clones contained pMal™-c2X (NewEnglandBiolabs) plasmids, over-expressing the following fusion proteins: Lane 1, maltose binding protein (MalE), lane 2, MalE-UU144, lane 3, MalE-UU171, lane 4, MalE-UU172N and lane 5, MalE-UU172C. Proteins were detected with hyperimmune antisera as indicated below the blots. As positive control, a hyperimmune antiserum was used that detected MalE and a variety of *E. coli* proteins.

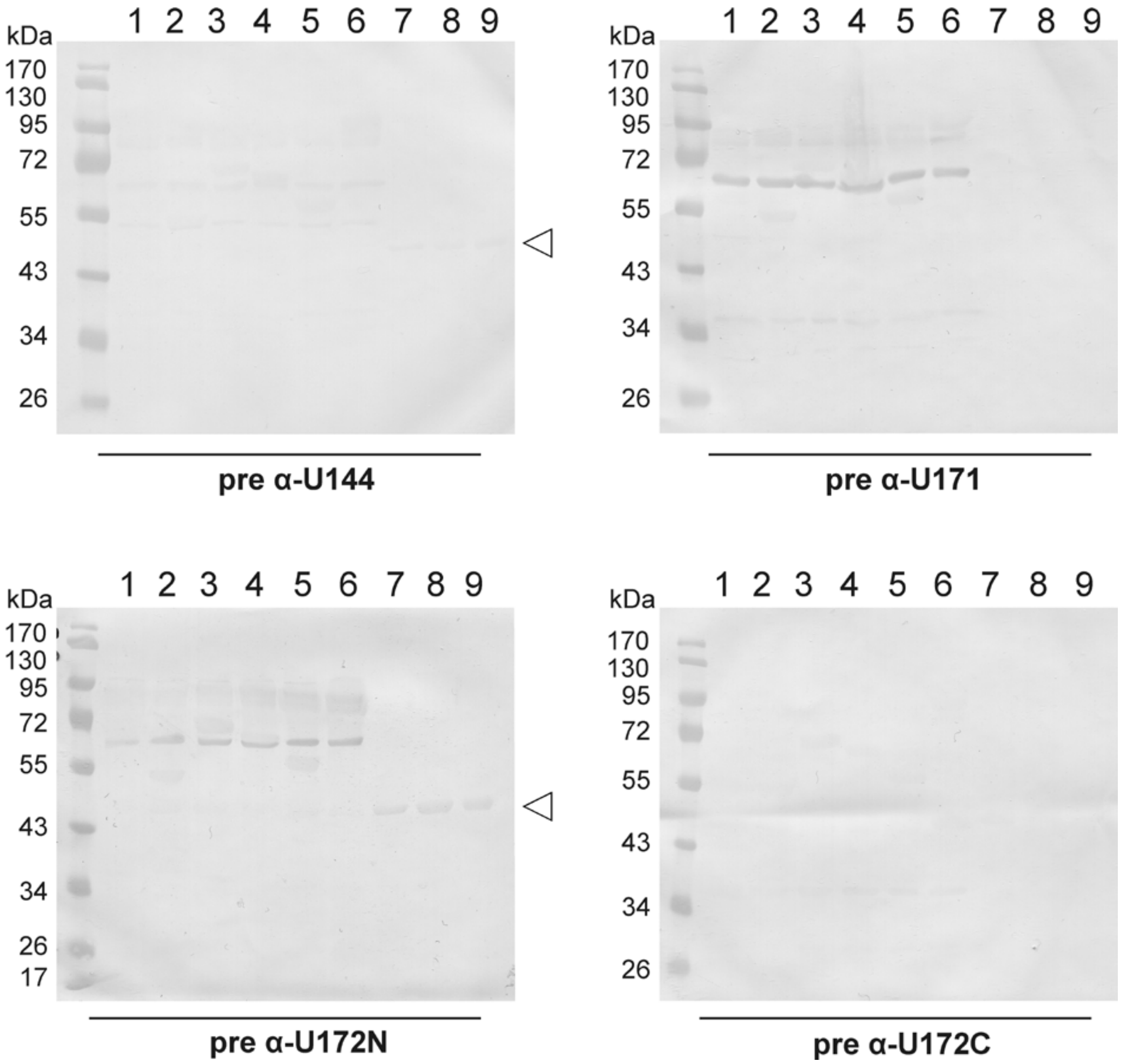

Fig. S6 Western blot analysis with pre-immune sera and total protein from *E. coli* clones and total *Ureaplasma* protein from *U. parvum* serovar 3 clonal variants.

E. coli clones contained pMal™-c2X (NewEnglandBiolabs) plasmids, over-expressing the following fusion proteins:

Lane 1, no over-expression, lane 2, maltose binding protein (MalE), lane 3, MalE-UU144, lane 4, MalE-UU171, lane 5, MalE-UU172N and lane 6, MalE-UU172C.

Total *Ureaplasma* protein was separated in lanes 7-9; lane 7, DR1-K1, lane 8, 27815^{T}-K5, and lane 9, M14-K16. Proteins were detected with antisera as indicated below the blots. Open triangle: background bands detecting protein from *Ureaplasma* protein preparations, which also appear in Western blots with monospecific polyclonal antibodies.
